# Trends in Liver Disease Etiology Among Adults Awaiting Liver Transplantation in the United States, 2014-2019

**DOI:** 10.1001/jamanetworkopen.2019.20294

**Published:** 2020-02-05

**Authors:** Robert J. Wong, Ashwani K. Singal

**Affiliations:** 1 Division of Gastroenterology and Hepatology, Alameda Health System, Highland Hospital, Oakland, California; 2 Sanford School of Medicine, University of South Dakota, Sioux Falls; 3 Avera Transplant Institute, Sioux Falls, South Dakota

## Abstract

This cohort study provides updated assessments of liver disease etiology trends among adults awaiting liver transplantation in the United States from 2014 to 2019.

## Introduction

Effective therapies for the hepatitis C virus (HCV) have transformed the landscape of chronic liver disease in the United States. Alcohol-associated liver disease (ALD) and nonalcoholic steatohepatitis (NASH) have emerged as significant clinical and economic health care burdens.^1,2^ Lack of effective therapies for ALD and NASH contribute to increasing disease severity among patients with these diseases, leading to cirrhosis and end-stage liver disease requiring liver transplantation (LT). In this cohort study, we updated assessments of liver disease etiology trends among adults awaiting LT in the United States.

## Methods

Using the United Network for Organ Sharing database from January 1, 2014, to March 31, 2019, this cohort study evaluated trends in liver disease etiology among adults registered for LT waiting lists in the United States. This study followed the Strengthening the Reporting of Observational Studies in Epidemiology (STROBE) reporting guideline. The Alameda Health System institutional review board granted exempt status and a waiver of informed consent given that the study used deidentified data from a large database and did not pose more than minimal risk.

Liver disease etiology was identified using United Network for Organ Sharing diagnosis codes.^3^ Among patients without hepatocellular carcinoma (HCC), etiology was determined with primary diagnosis codes. Among patients with HCC, secondary diagnosis codes were used to determine underlying liver disease etiology. In addition to separate diagnosis codes for HCV and ALD, the United Network for Organ Sharing includes codes for combined HCV/ALD; we included patients with primary HCV and secondary ALD or primary ALD and secondary HCV as HCV/ALD. Patients with NASH included those with NASH codes or cryptogenic cirrhosis based on previous studies.^3,4^ Annual liver disease etiology trends were calculated for the total wait-listed population and stratified by sex and self-reported race/ethnicity. Descriptive analyses were performed using Stata version 14.0 (StataCorp). No comparative statistical testing was performed, and thus, no threshold for statistical significance was set.

## Results

Among 51 329 adults registered to LT waiting lists from January 2014 to March 2019 (mean [SD] age, 56.8 [9.8] years; 17 861 [34.8%] women; 36 220 [71.7%] non-Hispanic white; 3866 [7.7%] African American; 8409 [16.6%] Hispanic; 2045 Asian [4.1%]), NASH and ALD became the leading liver disease etiologies (Figure). This increase was primarily seen among patients without HCC, and in the first quarter of 2019, ALD represented 40.3% (795 of 1974) and NASH represented 33.9% (669 of 1974) of wait-listed registrants without HCC (Table). The prevalence of HCV among patients with HCC declined, and in the first quarter of 2019, HCV represented 35.9% (177 of 493) and NASH represented 34.7% (171 of 493) of wait-listed registrants with HCC. Among women with HCC, NASH surpassed HCV in the second half of 2018 (NASH, 222 patients [41.0%]; HCV, 199 patients [36.7%]) and was the leading etiology among wait-listed registrants with and without HCC during the first quarter of 2019 (without HCC, 315 [39.1%]; with HCC, 58 [45.3%]). In contrast, ALD was the leading etiology among men without HCC in 2019 (ALD, 558 [47.7%]; NASH, 354 [30.3%]; HCV, 98 [8.4%]), whereas HCV, while declining, remained the leading etiology among men with HCC (HCV, 138 [37.8%]; NASH, 113 [31.0%]; ALD, 57 [15.6%]). Among patients without HCC, the leading etiology in 2019 was ALD among non-Hispanic white registrants (ALD, 634 [43.2%]; NASH, 499 [34.0%]; HCV, 100 [6.8%]), HCV and ALD among African American registrants (ALD, 28 [25.7%]; HCV, 22 [20.2%]; NASH, 12 [11.0%]), and NASH and ALD among Hispanic registrants (NASH, 119 [39.5%]; ALD, 106 [35.2%]; HCV, 26 [8.6%]); NASH, hepatitis B virus, and ALD were the leading etiologies among Asian registrants without HCC in 2019 (NASH, 18 [32.7%]; hepatitis B virus, 15 [27.3%]; ALD, 12 [21.8%]). Among patients with HCC, HCV declined but remained the leading etiology in 2019 among non-Hispanic white registrants (HCV, 119 [38.8%]; NASH, 110 [35.8%]; ALD, 40 [13.0%]) and African American registrants (HCV, 22 [56.4%]; NASH, 9 [23.1%]; ALD, 1 [2.6%]), whereas hepatitis B virus led among Asian registrants (hepatitis B virus, 19 [47.5%]; HCV, 10 [25.0%]; NASH, 8 [20.0%]). In the last quarter of 2018, NASH surpassed HCV as the leading etiology among Hispanic registrants with HCC (2016: NASH, 120 [26.4%]; HCV, 198 [43.5%]; 2018: NASH, 190 [35.1%]; HCV, 181 [33.4%]).

**Figure.**
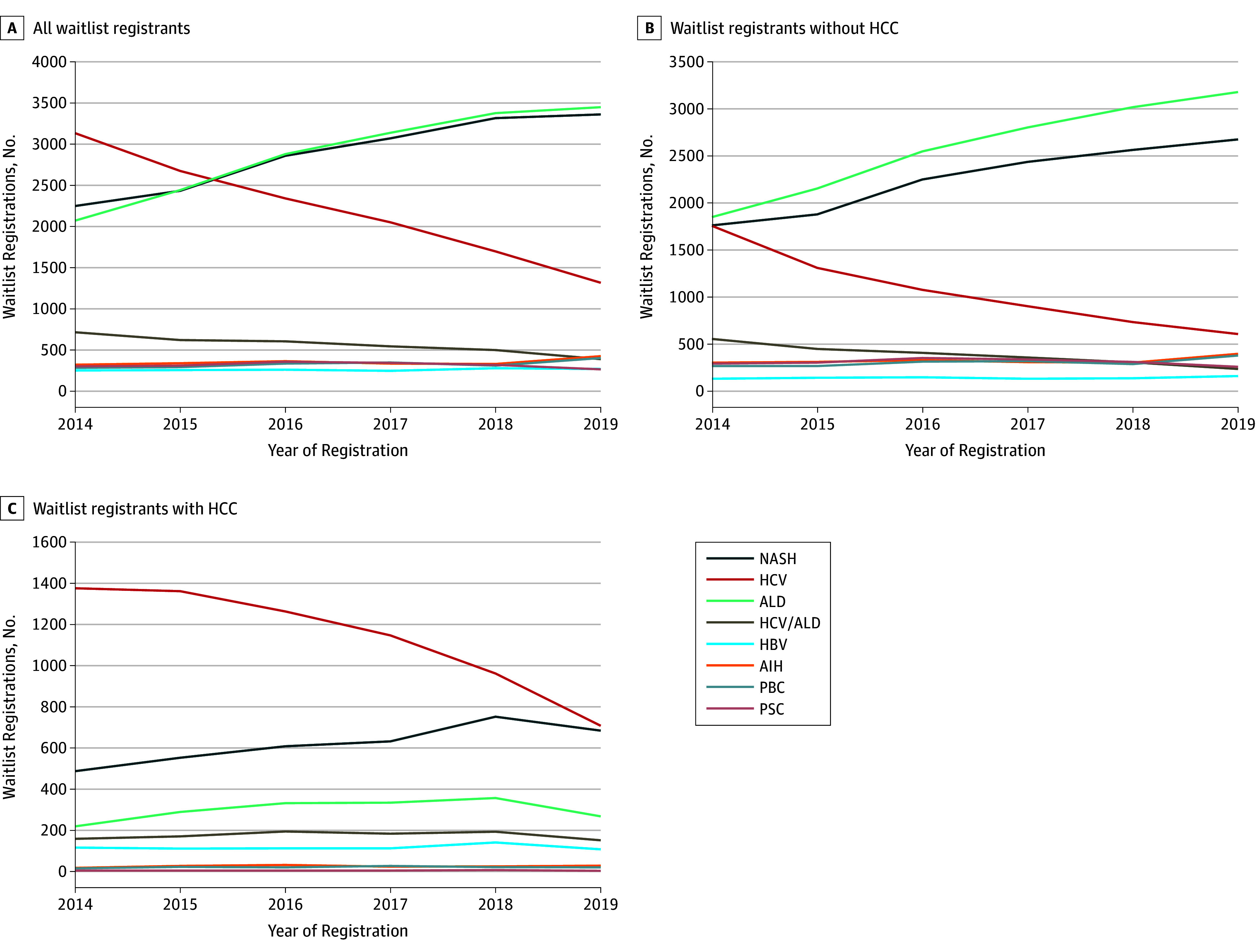
Liver Disease Etiology Trends Among Adult Liver Transplantation Waiting List Registrants in the United States Data for 2019 were available from January 1 to March 31, 2019. The total number of wait-listed registrants for 2019 was extrapolated by multiplying the numbers from January 1 to March 31 by 4. AIH indicates autoimmune hepatitis; ALD, alcohol-associated liver disease; HBV, hepatitis B virus; HCC, hepatocellular carcinoma; HCV, hepatitis C virus; NASH, nonalcoholic steatohepatitis; PBC, primary biliary cholangitis; and PSC, primary sclerosing cholangitis.

**Table.** Liver Disease Etiology Among Adults Registered to Liver Transplantation Waiting Lists With and Without HCC, by Sex and Race/Ethnicity

Group and Year	Patients, No. (%)							
	NASH	HCV	ALD	HCV/ALD	HBV	AIH	PBC	PSC
**Men**								
No HCC								
2014 (n = 4318)	911 (21.1)	1195 (27.7)	1356 (31.4)	437 (10.1)	100 (2.3)	79 (1.8)	45 (1.0)	195 (4.5)
2016 (n = 4542)	1173 (25.8)	731 (16.1)	1836 (40.4)	321 (7.1)	117 (2.6)	88 (1.9)	39 (0.9)	237 (5.2)
2018 (n = 4608)	1286 (27.9)	490 (10.6)	2120 (46.0)	237 (5.1)	105 (2.3)	90 (2.0)	44 (1.0)	236 (5.1)
2019 (n = 1169)^a^	354 (30.3)	98 (8.4)	558 (47.7)	49 (4.2)	29 (2.5)	23 (2.0)	12 (1.0)	46 (3.9)
HCC								
2014 (n = 1849)	321 (17.4)	1091 (59.0)	200 (10.8)	131 (7.1)	97 (5.2)	4 (0.2)	1 (0.1)	4 (0.2)
2016 (n = 1976)	427 (21.6)	977 (49.4)	292 (14.8)	171 (8.7)	96 (4.9)	5 (0.3)	4 (0.2)	4 (0.2)
2018 (n = 1916)	530 (27.7)	763 (39.8)	313 (16.3)	174 (9.1)	118 (6.2)	8 (0.4)	3 (0.2)	7 (0.4)
2019 (n = 365)^a^	113 (31.0)	138 (37.8)	57 (15.6)	35 (9.6)	19 (5.2)	2 (0.5)	0	1 (0.3)
**Women**								
No HCC								
2014 (n = 2603)	850 (32.7)	562 (21.6)	495 (19.0)	119 (4.6)	33 (1.3)	224 (8.6)	221 (8.5)	99 (3.8)
2016 (n = 2883)	1077 (37.4)	345 (12.0)	712 (24.7)	87 (3.0)	30 (1.0)	242 (8.4)	275 (9.5)	115 (4.0)
2018 (n = 3054)	1279 (41.9)	244 (8.0)	899 (29.4)	69 (2.3)	32 (1.0)	214 (7.0)	243 (8.0)	74 (2.4)
2019 (n = 805)^a^	315 (39.1)	54 (6.7)	237 (29.4)	10 (1.2)	11 (1.4)	76 (9.4)	83 (10.3)	19 (2.4)
HCC								
2014 (n = 547)	166 (30.3)	285 (52.1)	20 (3.7)	28 (5.1)	19 (3.5)	14 (2.6)	14 (2.6)	1 (0.2)
2016 (n = 593)	181 (30.5)	287 (48.4)	40 (6.7)	24 (4.0)	17 (2.9)	27 (4.6)	16 (2.7)	1 (0.2)
2018 (n = 542)	222 (41.0)	199 (36.7)	44 (8.1)	19 (3.5)	23 (4.2)	17 (3.1)	18 (3.3)	0
2019 (n = 128)^a^	58 (45.3)	39 (30.5)	10 (7.8)	3 (2.3)	8 (6.3)	5 (3.9)	5 (3.9)	0
**Non-Hispanic White**								
No HCC								
2014 (n = 5060)	1364 (27.0)	1190 (23.5)	1445 (28.6)	411 (8.1)	50 (1.0)	175 (3.5)	185 (3.7)	240 (4.7)
2016 (n = 5385)	1706 (31.7)	671 (12.5)	1985 (36.9)	282 (5.2)	48 (0.9)	198 (3.7)	221 (4.1)	274 (5.1)
2018 (n = 5637)	1952 (34.6)	484 (8.6)	2316 (41.1)	220 (3.9)	58 (1.0)	172 (3.1)	189 (3.4)	246 (4.4)
2019 (n = 1469)^a^	499 (34.0)	100 (6.8)	634 (43.2)	44 (3.0)	13 (0.9)	53 (3.6)	72 (4.9)	54 (3.7)
HCC								
2014 (n = 1531)	348 (22.7)	867 (56.6)	149 (9.7)	116 (7.6)	24 (1.6)	10 (0.7)	12 (0.8)	5 (0.3)
2016 (n = 1657)	444 (26.8)	799 (48.2)	215 (13.0)	139 (8.4)	22 (1.3)	23 (1.4)	12 (0.7)	3 (0.2)
2018 (n = 1461)	470 (32.2)	584 (40.0)	224 (15.3)	129 (8.8)	21 (1.4)	14 (1.0)	13 (0.9)	6 (0.4)
2019 (n = 307)^a^	110 (35.8)	119 (38.8)	40 (13.0)	27 (8.8)	3 (1.0)	4 (1.3)	3 (1.0)	1 (0.3)
**African American**								
No HCC								
2014 (n = 540)	43 (8.0)	251 (46.5)	69 (12.8)	47 (8.7)	11 (2.0)	64 (11.9)	20 (3.7)	35 (6.5)
2016 (n = 481)	53 (11.0)	168 (34.9)	87 (18.1)	32 (6.7)	18 (3.7)	57 (11.9)	26 (5.4)	40 (8.3)
2018 (n = 418)	58 (13.9)	101 (24.2)	114 (27.3)	26 (6.2)	15 (3.6)	53 (12.7)	17 (4.1)	34 (8.1)
2019 (n = 109)^a^	12 (11.0)	22 (20.2)	28 (25.7)	5 (4.6)	11 (10.1)	18 (16.5)	6 (5.5)	7 (6.4)
HCC								
2014 (n = 266)	25 (9.4)	209 (78.6)	4 (1.5)	10 (3.8)	13 (4.9)	5 (1.9)	0	0
2016 (n = 260)	23 (8.8)	190 (73.1)	5 (1.9)	23 (8.8)	12 (4.6)	6 (2.3)	0	1 (0.4)
2018 (n = 216)	45 (20.8)	129 (59.7)	11 (5.1)	12 (5.6)	12 (5.6)	4 (1.9)	2 (0.9)	1 (0.5)
2019 (n = 39)^a^	9 (23.1)	22 (56.4)	1 (2.6)	2 (5.1)	4 (10.3)	1 (2.6)	0	0
**Hispanic**								
No HCC								
2014 (n = 1004)	289 (28.8)	237 (23.6)	267 (26.6)	85 (8.5)	13 (1.3)	51 (5.1)	48 (4.8)	14 (1.4)
2016 (n = 1200)	390 (32.5)	184 (15.3)	390 (32.5)	86 (7.2)	11 (0.9)	61 (5.1)	56 (4.7)	22 (1.8)
2018 (n = 1261)	457 (36.2)	116 (9.2)	482 (38.2)	49 (3.9)	8 (0.6)	67 (5.3)	66 (5.2)	16 (1.3)
2019 (n = 301)^a^	119 (39.5)	26 (8.6)	106 (35.2)	9 (3.0)	1 (0.3)	22 (7.3)	16 (5.3)	2 (0.7)
HCC								
2014 (n = 419)	99 (23.6)	223 (53.2)	60 (14.3)	30 (7.2)	1 (0.2)	3 (0.7)	3 (0.7)	0
2016 (n = 455)	120 (26.4)	198 (43.5)	98 (21.5)	27 (5.9)	4 (0.9)	3 (0.7)	5 (1.1)	0
2018 (n = 542)	190 (35.1)	181 (33.4)	114 (21.0)	43 (7.9)	4 (0.7)	6 (1.1)	4 (0.7)	0
2019 (n = 100)^a^	44 (44.0)	23 (23.0)	24 (24.0)	7 (7.0)	1 (1.0)	0	1 (1.0)	0
**Asian**								
No HCC								
2014 (n = 217)	42 (19.4)	54 (24.9)	36 (16.6)	6 (2.8)	59 (27.2)	10 (4.6)	8 (3.7)	2 (0.9)
2016 (n = 245)	67 (27.3)	36 (14.7)	46 (18.8)	2 (0.8)	70 (28.6)	9 (3.7)	3 (1.2)	12 (4.9)
2018 (n = 207)	60 (29.0)	20 (9.7)	45 (21.7)	3 (1.4)	55 (26.6)	7 (3.4)	9 (4.3)	8 (3.9)
2019 (n = 55)^a^	18 (32.7)	3 (5.5)	12 (21.8)	0	15 (27.3)	4 (7.3)	1 (1.8)	2 (3.6)
HCC								
2014 (n = 153)	12 (7.8)	59 (38.6)	3 (2.0)	2 (1.3)	77 (50.3)	0	0	0
2016 (n = 164)	18 (11.0)	58 (35.4)	9 (5.5)	3 (1.8)	73 (44.5)	0	2 (1.2)	1 (0.6)
2018 (n = 204)	39 (19.1)	50 (24.5)	6 (2.9)	4 (2.0)	103 (50.5)	1 (0.5)	1 (0.5)	0
2019 (n = 40)^a^	8 (20.0)	10 (25.0)	1 (2.5)	1 (2.5)	19 (47.5)	1 (2.5)	0	0

Abbreviations: AIH, autoimmune hepatitis; ALD, alcoholic liver disease; HBV, hepatitis B virus; HCC, hepatocellular carcinoma; HCV, hepatitis C virus; NASH, nonalcoholic steatohepatitis; PBC, primary biliary cholangitis; PSC, primary sclerosing cholangitis.

^a^ Patients on waiting lists from January 1, 2019, to March 31, 2019.

## Discussion

This study showed that NASH and ALD have become the most common etiologies of liver disease among LT waiting list registrants without HCC, and NASH is becoming a leading indication in patients with HCC. This study has limitations, including potential misclassification bias in determining liver disease etiology among LT waiting list registrants.

While the declining prevalence of HCV among wait-listed registrants is a testament to the significant effect of HCV therapies in the United States, caution must be exercised in light of recent increases in HCV because of the opioid epidemic.^7^ Increases in the prevalence of NASH and ALD among registrants on LT waiting lists confirm the alarm previous studies have raised. Population-based studies have reported on the rising prevalence of alcoholic fatty liver disease and advanced fibrosis,^1^ the increasing prevalence of severe ALD with cirrhosis complications among hospitalized patients,^5^ and increasing cirrhosis death rates that are largely associated with alcoholic cirrhosis, particularly among individuals aged 25 to 34 years.^6^ While NASH therapies may be on the horizon, these data continue to highlight the urgent need to better address the dangers of unhealthy alcohol use from a public health perspective.
